# Craniofrontonasal dysplasia: A case report

**DOI:** 10.1002/pdi3.5

**Published:** 2023-06-10

**Authors:** Kanad Ghosh, Hope Xu, Mélissa Roy, Bakhtiar Yamini, Russell R. Reid

**Affiliations:** ^1^ Section of Plastic and Reconstructive Surgery Department of Surgery University of Chicago Chicago Illinois USA; ^2^ Department of Neurosurgery University of Chicago Chicago Illinois USA

**Keywords:** craniofrontonasal dysplasia, EFNB1, hypertelorism

## Abstract

Craniofrontonasal dysplasia (CFND) is a rare congenital malformation, which has a wide array of symptoms that can vary drastically between patients. These include coronal synostosis with associated brachycephaly, hypertelorism, cleft lip and palate, and limb malformations, among others. The pleomorphic nature of the disease and numerous clinical decisions required for its management present a unique challenge to craniofacial surgeons when considering indications and timing for surgical intervention. In this report, we present the case of a patient with CFND, their surgical management, and discuss updates in principles of management of CFND.

## 1 INTRODUCTION

Craniofrontonasal dysplasia (CFND) was first identified as a distinct diagnosis in a case report by Cohen in 1979, in which he discussed the occurrence of brachycephaly, hypertelorism, and various joint deformations in a mother and daughter.^1^ Since then, numerous published accounts have elucidated the genetics and presentation of this rare syndrome. In this report, we present the clinical course of a patient with CFND treated at our institution from presentation to surgical intervention. We will also discuss CFND from a symptomatic, genetic, and surgical perspective, while exploring recent advances in surgical principles and management.

## 2 CASE REPORT

**FIGURE 1:**
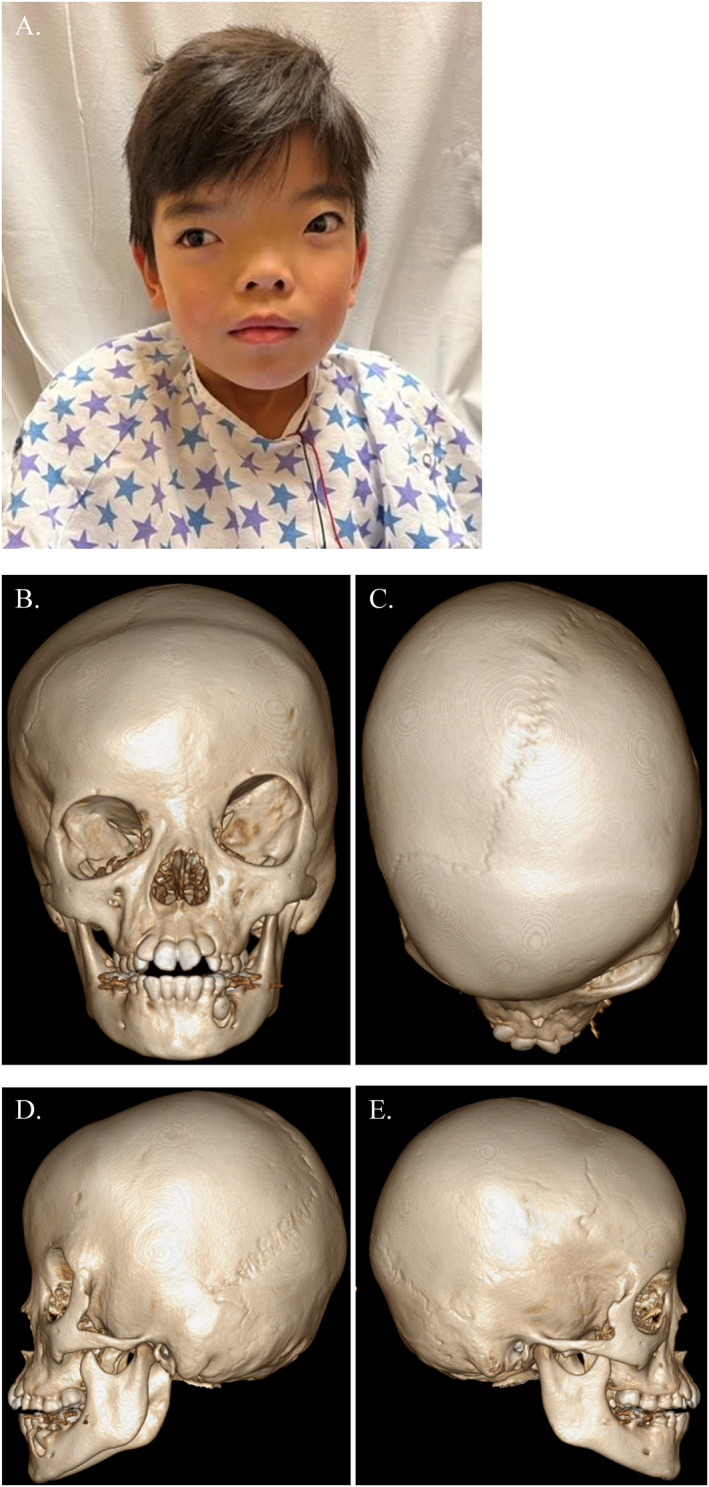
(A) Pre‐operative patient photo. (B–E) Pre‐operative three‐dimensional CT imaging.

Prior to the writing of the following Institutional Review Board (IRB) exempt case report, proper informed consent, including consent to use photographs, was obtained from the patient and family. A five‐year‐old female patient was presented to our institution for an initial clinical evaluation after having undergone international adoption (Figure 1). The patient had hypertelorism without ophthalmologic deficits and with normal fundoscopic examination. She had no developmental delay and was performing up to a grade level in school though her parents did endorse some behavioral issues. Subsequent CT imaging to evaluate the cranial vault showed no ventricular enlargement or mass effect. Her initial head circumference was 48 cm with a medial intercanthal distance (MICD) of 48 mm. She was noted to have left coronal suture ridging, contralateral frontal bossing, and rightward chin point deviation with an anterior open bite. There was no cleft lip or palate. At the time, it was decided to postpone surgical correction of hypertelorism until she was older. She continued yearly follow‐up until the age of eight, at which time her parents agreed to proceed with the correction of her craniosynostosis and hypertelorism with fronto‐orbital advancement (FOA) and bilateral box osteotomies.

**FIGURE 2:**
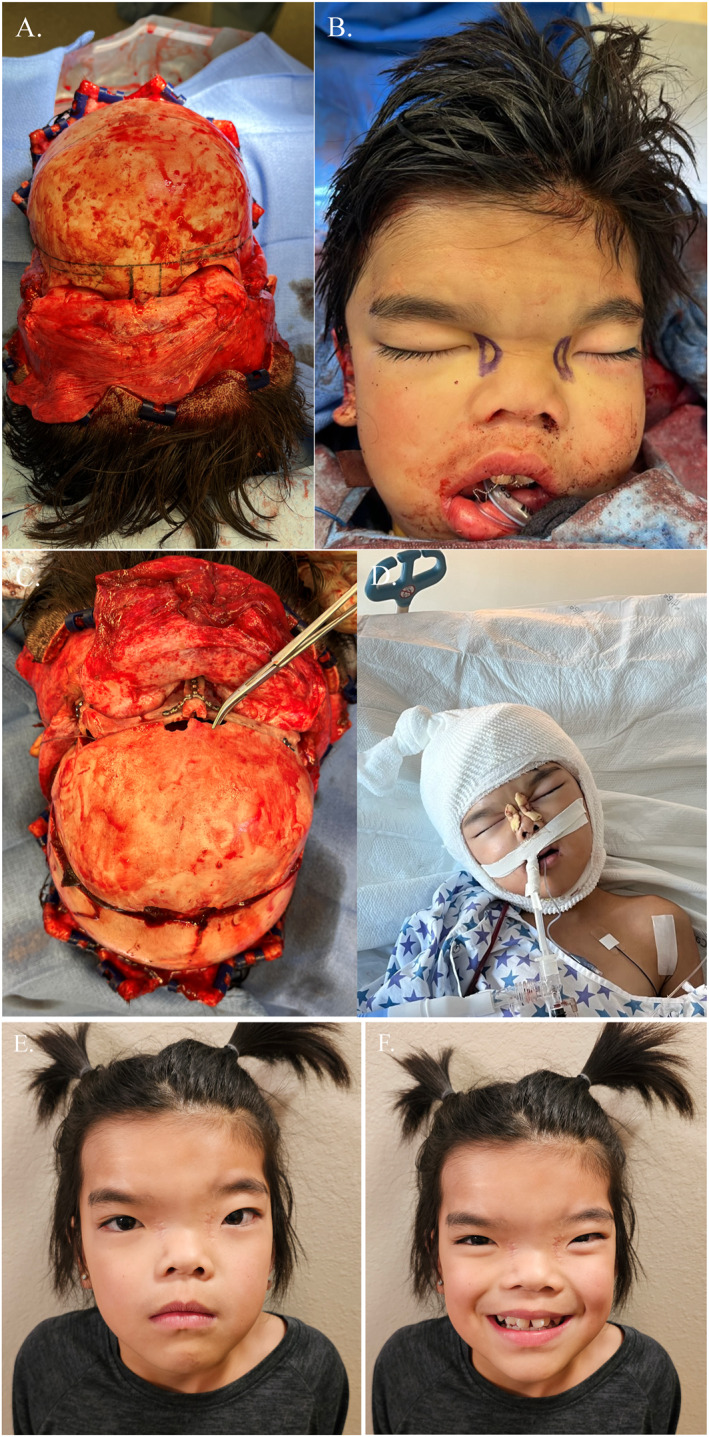
(A) Intraoperative photo following pericranial flap elevation showing markings for fronto‐orbital advancement. (B) Intraoperative box osteotomy markings. (C) Intraoperative photo following fronto‐orbital advancement and box osteotomy with plate fixation in place. (D) Immediate post‐operative photo. (E–F) Six month post‐operative photos.

Preoperatively, computer‐assisted surgical planning (CASP) was implemented to fabricate 3D‐printed marking and cutting guides for extracranial and intracranial osteotomies (Figure 2A–D). Craniotomy and exposure of the anterior cranial base were performed by neurosurgery, after which the plastic surgery team performed asymmetric orbital box osteotomies due to the patient's pre‐existing orbital asymmetries. The orbital boxes were medialized and the excised frontonasal bone was converted to nasal cantilever bone graft, followed by reconstruction of the bilateral tenon area with interpositional bone grafts. The bifrontal craniotomy construct was rotated 180° and advanced to improve contour of the forehead along with the advancement of the left hemi‐orbit. Finally, medial canthopexies were performed, extraocular movement was confirmed with forced duction, and the scalp was closed in a layered fashion. The patient was admitted to the Pediatric ICU for close monitoring. Postoperatively, she recovered appropriately without transfusion requirement and was discharged on post‐operative day 3. A 6‐month follow‐up (Figure 2) demonstrates stable fronto‐orbital advancement and improvement in orbital positioning.

## 3 DISCUSSION

The varied clinical presentation of CFND patients complexifies diagnostic accuracy. The most common findings include hypertelorism, telecanthus, deficient nasal tip with broadened nasal bridge, and brachycephaly. Other common findings include cleft lip and palate, high arched palate, syndactyly of the fingers and toes, abnormalities of the hips and shoulder girdle, frizzy hair, and nail grooving.^2^ One of the earliest observations was a sex‐specific pattern of presentation with females having higher rates of craniosynostosis, neck webbing, and limb and trunk malformations. Genital and thoracic abnormalities, including congenital diaphragmatic hernia, have been seen in both males and females.^3^, ^4^, ^5^, ^6^, ^7^ This correlated with a sex‐linked mode of inheritance with both daughters and sons of affected mothers and daughters of affected fathers presenting with CFND symptoms, but no reports of sons of affected fathers having any CFND‐related abnormalities.^8^ The aberrant gene in CFND was later determined to be EFNB1 on the X chromosome, which encodes for a bidirectional signaling protein named ephrin B1 that acts as both a receptor and a ligand affecting cell migration during morphogenesis and neuronal growth.^3^ The unusual severity of this X‐linked disorder in heterozygous females as opposed to hemizygous males has been attributed to cellular interference and X inactivation, whereby the presence of normal ephrin B1 in a subset of cells leads to binding with non‐ephrin ligands and disturbs normal compartmentalized signaling pathways.^6^

Though there have been several reports of surgical techniques and outcomes, there are few defined recommended approaches for management of CFND. Kawamoto and colleagues established a treatment algorithm for CFND both based on timing of presentation and severity of disease.^9^ Based on their results from 21 patients, they recommend early FOA for craniosynostosis, ideally at 6 months but preferably before four‐years‐old. In patients presenting after 4 years of age, FOA is deferred until the time of hypertelorism correction. Principles behind hypertelorism management using both intracranial and extracranial approaches were first established by Tessier in 1974, who also developed a classification system based on inter‐dacryon distance (first degree: 30–34 mm, second degree: 34–40 mm, third degree: >40 mm).^10^ Orbital box osteotomies, described in the case above, allows for movement of the orbits in both the horizontal and vertical planes and can account for baseline asymmetry pre‐operatively. Facial bipartition was first described by Van der Meulen in 1979. It allows for the correction of hypertelorism and maxillary deformities, such as high‐arched palate via extension of bony partition through the hard palate. It also decreases the risk of injury to developing tooth buds. Though indications for either technique vary, there is no consensus on the respective long‐term recurrence rates of bony hypertelorism.

Investigations on CFND ranging from cellular mechanisms to genetics and surgical techniques are still ongoing. Advances in CASP and intraoperative navigation have allowed greater control and foresight regarding surgical management of CFND and other complex craniofacial deformities. Still, there remains a dearth of large‐scale, long‐term data to accurately determine the best timing and methods of surgical intervention and their associated outcomes.

## AUTHOR CONTRIBUTIONS

**Kanad Ghosh**: Chart review, literature review on CFND and manuscript writing. **Hope Xu**: Chart review, literature review on CFND and manuscript writing. **Melissa Roy**: Manuscript review and revisions. **Bakhtiar Yamini**: Senior neurosurgeon involved in case; manuscript review and revisions. **Russell R. Reid**: Concept development of manuscript; senior plastic surgeon involved in case; photo and figure procurement; manuscript review and revisions.

## CONFLICT OF INTEREST STATEMENT

Russell R. Reid is the member of the *Pediatric Discovery* Editorial Board. To minimize bias, he was excluded from all editorial decision‐making related to the acceptance of this article for publication. The remaining authors declare no conflict of interest.

## ETHICS STATEMENT

Prior to the writing of the following Institutional Review Board (IRB) exempt case report, proper informed consent, including consent to use photographs, was obtained from the patient and family.

## Data Availability

The data that support the findings of this study are available on request from the corresponding author. The data are not publicly available due to privacy or ethical restrictions.

## References

[1] Cohen MM, Jr . Craniofrontonasal dysplasia. Birth Defects Orig Artic Ser. 1979;15(5B):85‐89. 526593

[2] Orr DJ , Slaney S , Ashworth GJ , Poole MD . Craniofrontonasal dysplasia. Br J Plast Surg. 1997;50(3):153‐161. 9176000 10.1016/s0007-1226(97)91362-x

[3] Zafeiriou DI , Pavlidou EL , Vargiami E . Diverse clinical and genetic aspects of craniofrontonasal syndrome. Pediatr Neurol. 2011;44(2):83‐87. 21215906 10.1016/j.pediatrneurol.2010.10.012

[4] Devriendt K , Van Mol C , Fryns JP . Craniofrontonasal dysplasia: more severe expression in the mother than in her son. Genet Couns. 1995;6(4):361‐364. 8775424

[5] Wieacker P , Wieland I . Clinical and genetic aspects of craniofrontonasal syndrome: towards resolving a genetic paradox. Mol Genet Metab. 2005;86(1‐2):110‐116. 16143553 10.1016/j.ymgme.2005.07.017

[6] Morris CA , Palumbos JC , Carey JC . Delineation of the male phenotype in carniofrontonasal syndrome. Am J Med Genet. 1987;27(3):623‐631. 3631134 10.1002/ajmg.1320270315

[7] Vasudevan PC , Twigg SR , Mulliken JB , Cook JA , Quarrell OW , Wilkie AO . Expanding the phenotype of craniofrontonasal syndrome: two unrelated boys with EFNB1 mutations and congenital diaphragmatic hernia. Eur J Hum Genet. 2006;14(7):884‐887. 16639408 10.1038/sj.ejhg.5201633

[8] Kapusta L , Brunner HG , Hamel BC . Craniofrontonasal dysplasia. Eur J Pediatr. 1992;151(11):837‐841. 1468459 10.1007/BF01957936

[9] Kawamoto HK , Heller JB , Heller MM , et al. Craniofrontonasal dysplasia: a surgical treatment algorithm. Plast Reconstr Surg. 2007;120(7):1943‐1956. 18090758 10.1097/01.prs.0000287286.12944.9f

[10] Tessier P . Experiences in the treatment of orbital hypertelorism. Plast Reconstr Surg. 1974;53(1):1‐18. 4588596 10.1097/00006534-197401000-00001

